# Topological transitions in ac/dc﻿-driven superconductor nanotubes

**DOI:** 10.1038/s41598-022-13543-0

**Published:** 2022-06-16

**Authors:** Vladimir M. Fomin, Roman O. Rezaev, Oleksandr V. Dobrovolskiy

**Affiliations:** 1 Institute for Integrative Nanosciences, Leibniz IFW Dresden, Helmholtzstraße 20, 01069 Dresden, Germany; 2 Laboratory of Physics and Engineering of Nanomaterials, Department of Theoretical Physics, Moldova State University, strada A. Mateevici 60, 2009 Chisinau, Republic of Moldova; 3 Institute of Engineering Physics for Biomedicine, National Research Nuclear University “MEPhI”, Kashirskoe shosse 31, Moscow, 115409 Russia; 4 Tomsk Polytechnic University, Lenin av. 30, Tomsk, 634050 Russia; 5 University of Vienna, Faculty of Physics, Nanomagnetism and Magnonics, Superconductivity and Spintronics Laboratory, Währinger Str. 17, 1090 Vienna, Austria

**Keywords:** Nanoscience and technology, Physics

## Abstract

Extending of nanostructures into the third dimension has become a major research avenue in condensed-matter physics, because of geometry- and topology-induced phenomena. In this regard, superconductor 3D nanoarchitectures feature magnetic field inhomogeneity, non-trivial topology of Meissner currents and complex dynamics of topological defects. Here, we investigate theoretically topological transitions in the dynamics of vortices and slips of the phase of the order parameter in open superconductor nanotubes under a modulated transport current. Relying upon the time-dependent Ginzburg–Landau equation, we reveal two distinct voltage regimes when (i) a dominant part of the tube is in either the normal or superconducting state and (ii) a complex interplay between vortices, phase-slip regions and screening currents determines a rich FFT voltage spectrum. Our findings unveil novel dynamical states in superconductor open nanotubes, such as paraxial and azimuthal phase-slip regions, their branching and coexistence with vortices, and allow for control of these states by superimposed dc and ac current stimuli.

## Introduction

Three-dimensional (3D) nanoarchitectures have become of increasing importance across various domains of science and technology^1–3^. They attract great attention in semiconductor physics^4,5^, magnetism^6,7^, photonics^8^, magnonics^9^, plasmonics^10^ and superconductivity^11^. The roll-up technology^12^ and direct-write nanoprinting using focused particle beams^13^ allow the realization of various complex-shaped geometries, appealing for investigations of their electronic, optical, magnetic and transport properties, and the development of novel applications. From a holistic point of view, geometry- and topology-induced phenomena in 3D nanoarchitectures have recently been analyzed for curvilinear semiconductor, superconductor, and magnetic nanoarchitectures, as well as for catalytic tubular micromotors and optical waveguides^1,2^.

In superconductivity, the hybridization of curved geometry with non-trivial topology is an established source of emerging physics^14–22^. Thus, self-rolled nanomembranes^23–30^ and direct-write 3D nanoarchitectures^31,32^ are interesting platforms for the examination of theoretical models and the experimental exploration of the intertwined dynamics of Meissner currents and *topological defects* (Abrikosov vortices and phase slips) of the order parameter in superconductors. From the viewpoint of applications, the extension of nanoscale superconductors into the third dimension allows for the full-vector-field sensing in quantum interferometry^33^, noise-equivalent power reduction in bolometry^28^ and reduction of footprints of fluxonic devices^32,34^. In this regard, magnetic flux transport at large dc currents^35–38^, GHz ac frequencies^39–42^ and in connection with optical/infrared-range photon absorption^43,44^ appears especially interesting for applications.

The motion of vortices under a high-frequency ac drive exhibits a rich variety of dynamics regimes which are determined by both, the ac amplitude and the ac frequency. Distinct from the translatory vortex motion under a dc current drive^45^, an ac current causes oscillatory motion of vortices^39,46^. However, the evolution of the superconducting state in 3D micro- and nanostructures has so far been studied in the regimes of dc or ac currents separately^28,31,32,47^. Though, from previous studies of planar films it is known that a combination of dc and ac stimuli can give rise to novel phenomena, such as dc/ac quantum interference^48,49^, rectified voltage and its reversal^50,51^, and peculiarities in the microwave power absorption^42^. Accordingly, (dc+ac)-driven curved 3D superconductor nanoarchitectures are expected to harbor novel physical phenomena which may possess potential for applications.

Here, we predict novel patterns of vortices and phase slips, and transitions between them, in (dc+ac)-driven open nanotubes under normal-to-tube-axis magnetic fields. The topological transitions are analyzed theoretically, relying upon the time-dependent Ginzburg–Landau (TDGL) equation. The revealed patterns include phase-slip regions extending *along* the transport current direction, their *branching* and *coexistence* with Abrikosov vortices. We identify two qualitatively different regimes in the voltage response which can be accessed experimentally. The first regime features a pronounced first harmonic in the fast Fourier transform (FFT) spectrum of the induced voltage. This regime occurs when the dominant area of the open tube is in the superconducting or normal state. The second regime entails a rich FFT spectrum of the induced voltage, because of the complex interplay between the dynamics of vortices and phase slips and the dynamics of the screening currents. Our findings shed light on the spatiotemporal evolution of the superconducting order parameter in open nanotubes and allow for its control via the induced voltage.

## Results

### Investigated system

**Figure 1:**
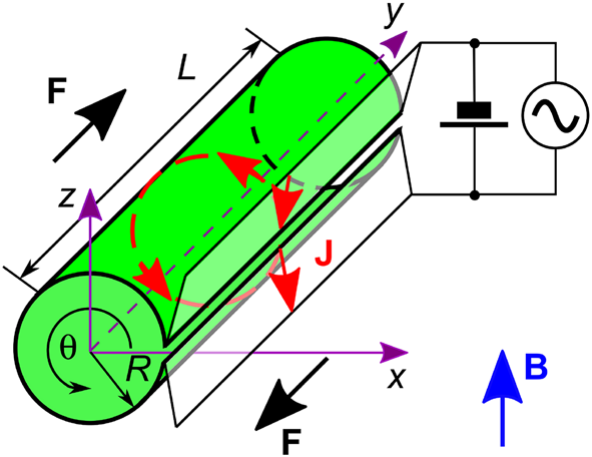
Geometry of the system. An open superconducting nanotube is in a magnetic field with induction ${\mathbf {B}}$ directed perpendicular to the tube axis. A combination of the dc and ac transport currents ${\mathbf {J}}$ flows along the generatrix of the tube and exerts the driving force ${\mathbf {F}}$ on superconducting vortices. The voltage associated with the dynamics of topological defects in the tube is measured between two electrodes attached at both sides of the slit.

We consider an open superconductor tube with length $L = 5\,\upmu \hbox {m}$ and radius $R = 400\,\hbox {nm}$. The tube is supposed to be made from a 50 nm-thick Nb film. Such tubes can be fabricated by the roll-up technology^52,53^. The geometry of the considered system is shown in Fig. 1 and the tube parameters are detailed in Table 1. Two electrodes are attached to the slit edges in order to apply a transport current. The width of the slit $\delta$ is supposed to be much smaller than the circumference $2\pi R$. The electrodes extend through the entire slit edges. The tube is in the magnetic field ${\mathbf {B}} = B{\mathbf {e}}_z$, which induces Meissner currents circulating within each half-tube^23^.

The temperature is taken $T=0.77\,T_{\mathrm {c}}$, where $T_{\mathrm {c}}$ is the critical temperature of the Nb film. This temperature is chosen as a trade-off between the regime where the use of the TDGL equation is justified ($T\rightarrow T_{\mathrm {c}}$) and the low-temperature regime with a larger variation of the superconducting order parameter.

In the presence of a transport current density $j_{\mathrm {tr}}(t) = j_0 + j_1\sin (2\pi ft)$ [$j_0$: dc current density, $j_1$ and *f*: ac current density amplitude and frequency], the dynamics of the order parameter in the tube reveals a modulation reflecting the different patterns of topological defects and transitions between them. Next, we outline the key stages of the evolution of the order parameter and consider the effects of each of the driving parameters on the patterns of topological defects therein.

### Time evolution of the order parameter and the induced voltage

**Figure 2:**
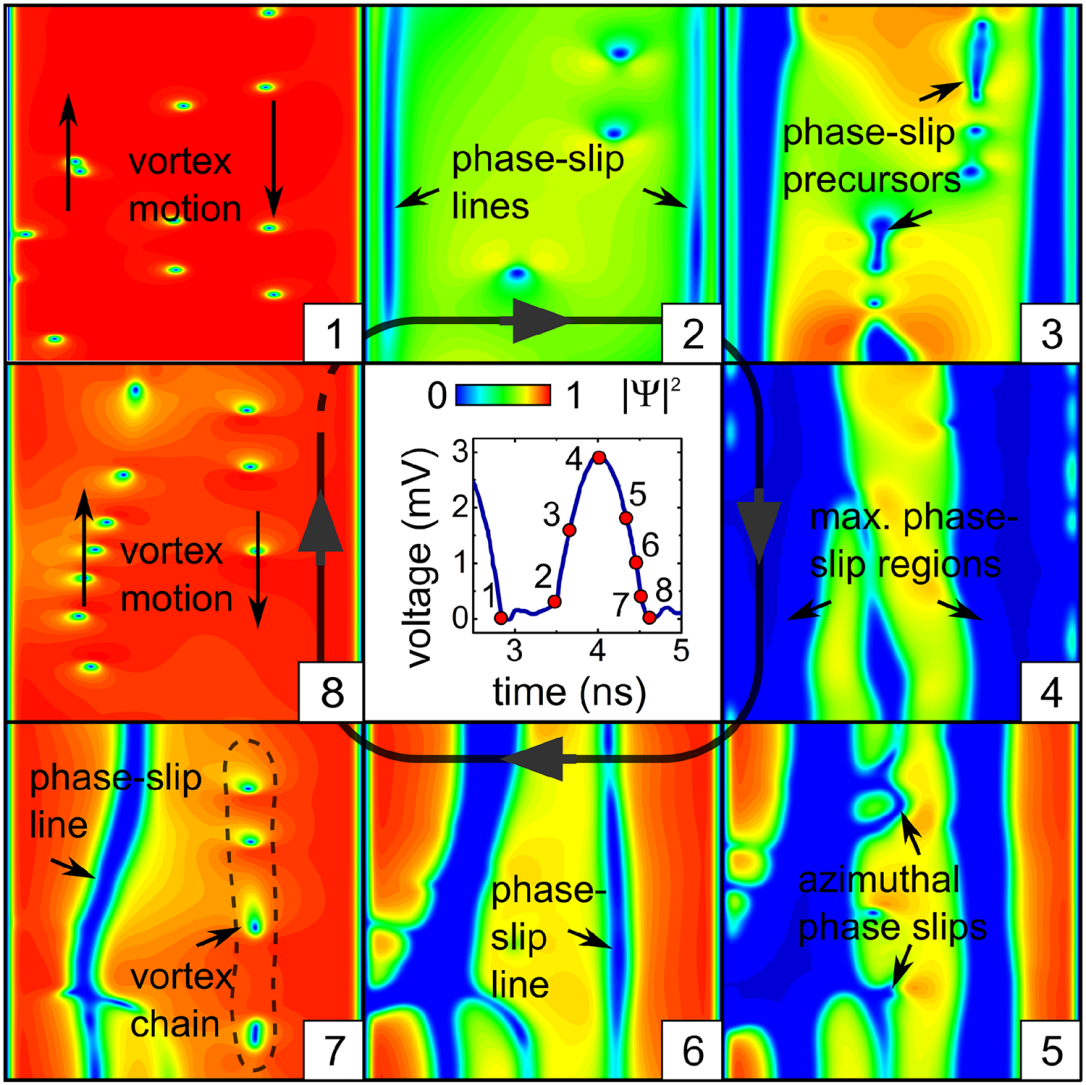
Dynamics of topological defects in the low-frequency regime. Evolution of the modulus of the order parameter for the dc density $j_0 = 2.1$ $\hbox {GAm}^{-2}$, the current modulation depth $j_1/j_0=0.5$ and ac frequency $f = 0.6$ GHz at $B=2$ mT. The sizes of all panels (height $\times$ width) are equal to $L \times 2\pi R$ (length $\times$ circumference of the tube). The directions of the vortex motion in the half-tubes are indicated by the arrows.The central panel represents the time-dependent voltage *U*(*t*).

Figure 2 presents the snapshots of the order parameter $|\Psi |^2$ on the tube surface at time points 1 to 8 of one ac cycle, as indicated in the middle panel. The plots are calculated for the dc density $j_0 = 2.1$ $\hbox {GAm}^{-2}$, the current modulation depth $j_1/j_0=0.5$ and the ac frequency $0.6\,$ GHz at $B=2$ mT. The modulation depth $j_1/j_0=0.5$ makes accessible both subcritical and overcritical regimes with respect to the total current but, at the same time, it is not yet large enough to allow the total current to change its polarity at the negative ac halfwave. Supplementary Video 1 presents the time evolution of the modulus and phase of the order parameter, the electric potential, and the time-dependent voltage *U*(*t*).

The superconducting order parameter developing from a random initial state, after some relaxation, reaches a quasi-stationary state which evolves nearly periodically, see Fig. 2. The key stages of this evolution are summarized next.

(1) At the smallest $j_{\mathrm {tr}}$, there are a few vortices moving in opposite directions in the two half-tubes. The induced voltage *U* is close to zero. (2) Two phase-slip regions appear close to the slit banks. The induced voltage slightly increases. (3) The phase-slip regions extend from the slit banks and induce a notable voltage drop. Precursors of further phase slips appear in the central regions of both half-tubes as well as in the opposite-to-slit region. The voltage increases further. (4) At the largest $j_{\mathrm {tr}}$, the phase slips from stage 2 reach their maximal sizes, whereas the phase slips from stage 3 fade out. The voltage is maximal. With a decrease of $j_{\mathrm {tr}}$, the order parameter begins to increase first in the opposite-to-slit region.

(5) New azimuthal phase-slip regions are developing between the paraxial phase-slip regions. The phase-slip regions of regime 2 depart from the slit banks and shrink. The voltage decreases. (6) The phase slips of both types coexist. The phase-slip regions further shrink to phase-slip lines. (7) The phase-slip lines split into vortex chains. The voltage decreases to almost zero. (8) After completion of one ac period, again only two chains of vortices moving in opposite directions in the two half-tubes are present, and the voltage is close to zero.

The time-dependent voltage *U*(*t*) has a rich FFT spectrum, which indicates that the induced voltage is a nonlinear function of the transport current. In the majority of studied cases, the first harmonic of the modulation depth of *U*(*t*) is at least by an order of magnitude larger than the others, while the second and third harmonics are comparable with each other (see Table SI-1 for details).

**Figure 3:**
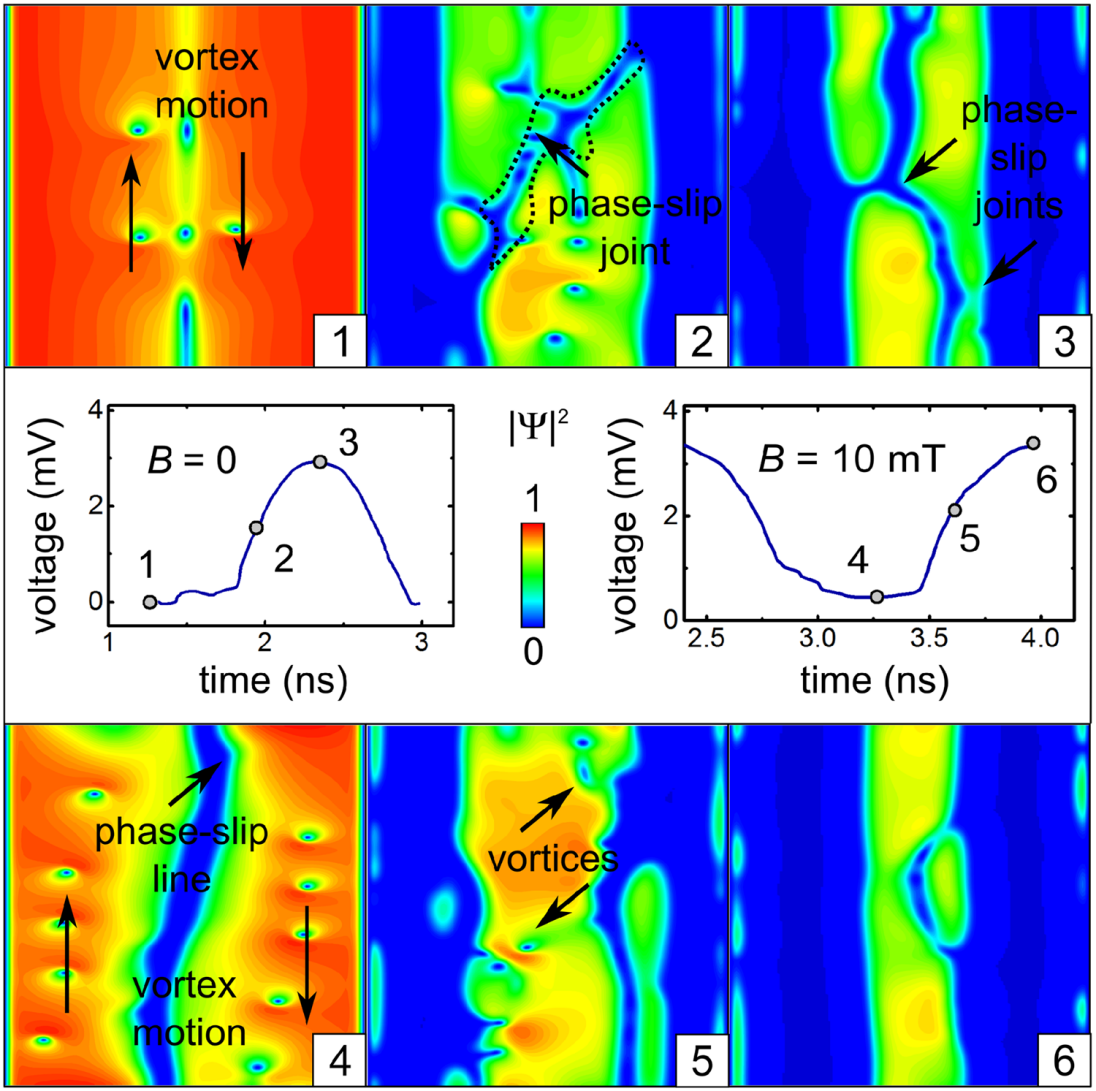
Effect of the magnetic field on the dynamics of topological defects. Evolution of the modulus of the order parameter and for $B =0$ (upper row) and $B=10\,\hbox {mT}$ (bottom row) at $j_0 = 2.1\,\hbox {GAm}^{-2}$, $j_1/j_0=0.5$ and $f = 0.6\,\hbox {GHz}$. The time-dependent induced voltage is shown in the middle row.

### Effect of the magnetic field at low ac frequencies

In zero magnetic field (Fig. 3, upper row), the key difference from the just considered case of $B=2\,\hbox {mT}$ consists in that (1) vortices do not move in some ordered way at the weakest $j_{\mathrm {tr}}$, so that the minimum value of *U*(*t*) is zero. The vortex nucleation is facilitated in the region with a weaker superconductivity, which at $t = 1.32\,\hbox {ns}$ is just the vicinity of the line opposite to the slit. Here, a parallel can be drawn with 2D planar strips in which the first phase-slip region appears in the middle because of the symmetry reasons^54^. With further evolution, an increase of the transport current density $j_{\mathrm {tr}}$ leads to a widening of the phase slips near the slit banks and to the occurrence of new phase slips in the opposite-to-slit region (2). At a larger $j_{\mathrm {tr}}$, a further phase slip can emerge (3), which joins both halves of the tube. This suggests that we deal with the states of holistic nature, which belong to the entire superconductor open tube, rather than to its halves separately.

At $B=10$ mT (Fig. 3, bottom row) and the weakest $j_{\mathrm {tr}}$, two vortex chains coexist with a phase-slip line in the opposite-to-slit region (4). With increasing $j_{\mathrm {tr}}$, the vortex chains evolve into phase-slip regions which grow (5) and tend to get interconnected (6). Interestingly, while the vortices in the major phase-slip areas move in the paraxial direction, the interconnects correspond to vortices moving predominantly in the azimuthal direction. This trend develops gradually with increasing magnetic field from 2 to 6 mT. The evolution of patterns of topological defects at different magnetic fields is detailed in Supplementary Video 1.

**Figure 4:**
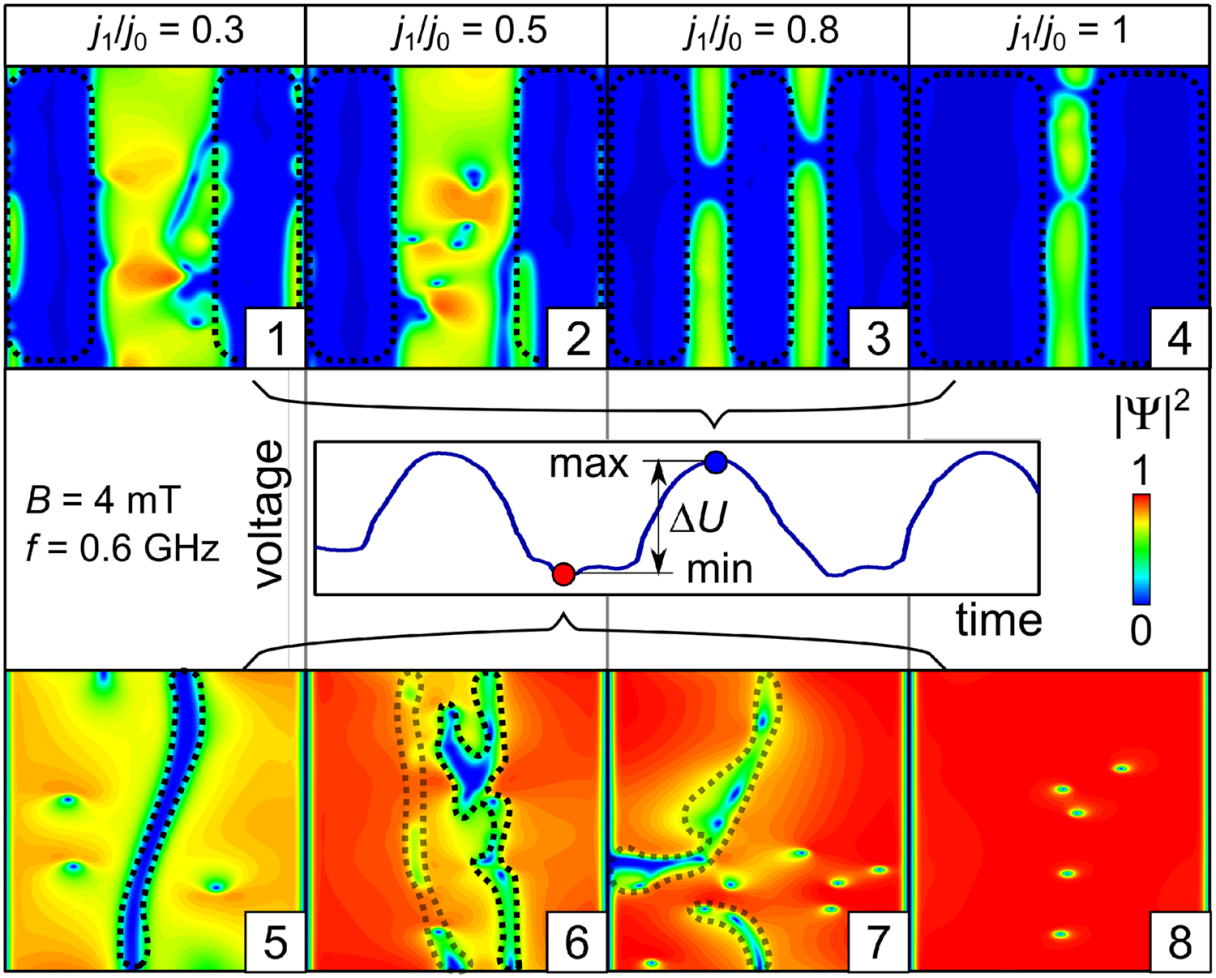
Effect of the current modulation depth $j_1/j_0$ on the the dynamics of topological defects. The time-dependent induced voltage is shown in the middle row. Patterns of the order parameter at the maximal and minimal values of the induced voltage *U*(*t*) at $B = 4\,\hbox {mT}$ $j_0 = 2.1\,\hbox {GAm}^{-2}$ and $f=0.6\,\hbox {GHz}$. The dashed lines encage the phase-slip regions.

### Effect of the current modulation depth

Figure 4 shows the effect of the current modulation depth $j_1/j_0$ on the patterns of $|\Psi |^2$ at the maximal and minimal values of the induced voltage *U*(*t*) at $B = 4\,\hbox {mT}$ $j_0 = 2.1\,\hbox {GAm}^{-2}$ and $f=0.6$ GHz. The typical time voltage signal is shown in the middle panel of Fig. 4. At $j_1/j_0=0.3$, the minimal voltage corresponds to state (5) with two vortex chains in each half-tube, which are separated by a paraxial phase-slip line in the opposite-to-slit region. The maximal voltage is induced by state (1) featuring two phase-slip regions close to the slit edges.

With increase of $j_1/j_0$, the state corresponding to the minimal voltage evolves as follows: the vortex chains disappear and a complex-shaped phase-slip region appears close to the opposite-to-slit region (6), the phase-slip region splits up into azimuthal and paraxial sections which are accompanied by a few vortices (7), the phase slips disappear and the voltage response is mediated by Abrikosov vortices (8). The evolution of the state corresponding to the maximal voltage includes the appearance of vortices and branching of the phase-slip areas close to the opposite-to-slit region (2) and a topological transition between the states with three (3) and two (4) phase-slip regions. The time evolution of the order parameter and the induced voltage is illustrated in Supplementary Video 2.

**Figure 5:**
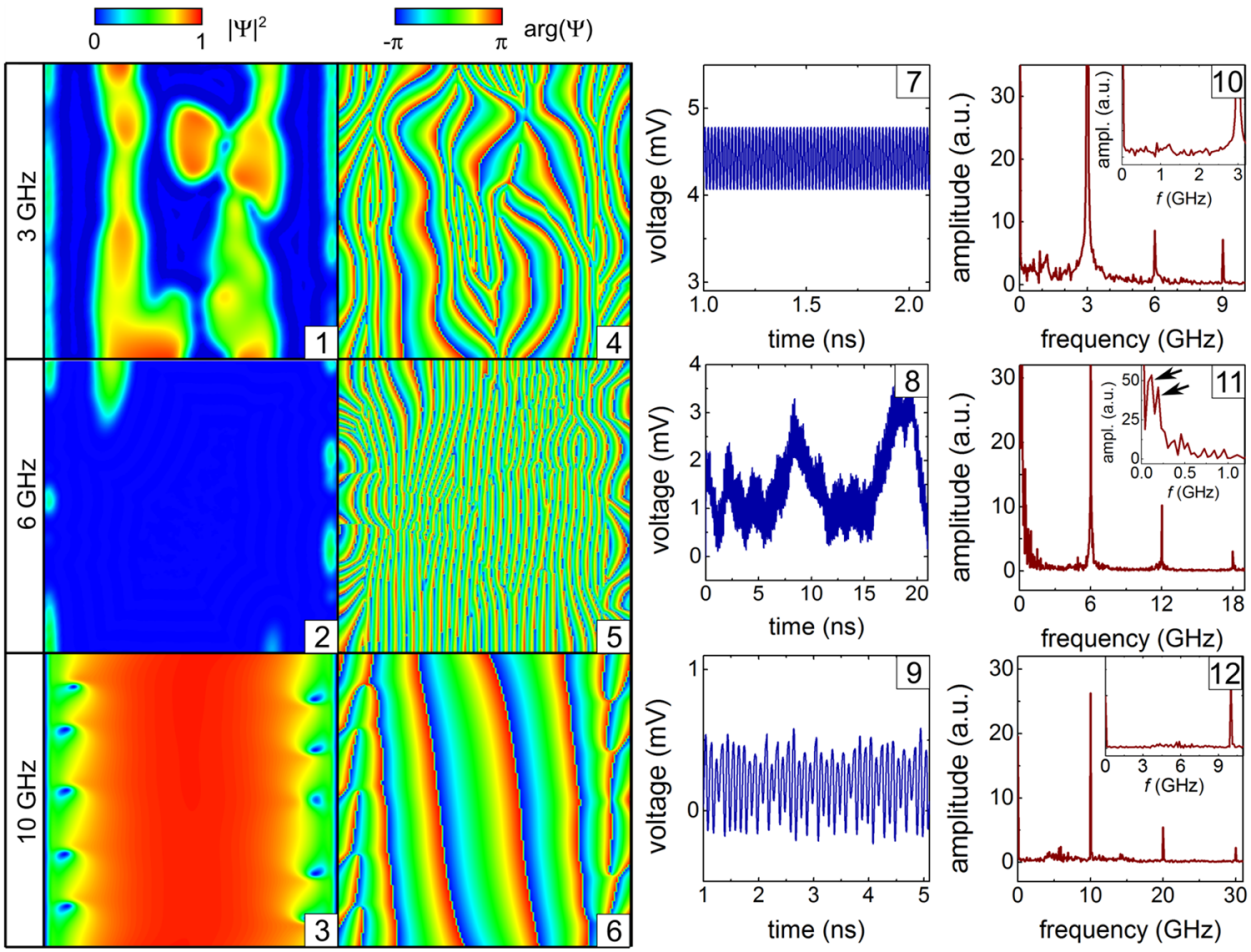
Effect of the ac frequency on the dynamics of topological defects. Modulus (panels 1–3) and phase (panels 4–6) of the order parameter at the maxima of the time-dependent voltage (panels 7–9) and its FFT spectra (panels 10–12) at $B= 2\,\hbox {mT}$, $j_0 = 2.1\,\hbox {GAm}^{-2}$ and $j_1/j_0 = 0.5$ for the ac frequencies 3, 6, and 10 GHz. The second- and third-highest peaks are indicated by arrows in the inset of panel (11).

### Effect of the ac frequency

The main effect of the ac frequency (Fig. 5) consists in the realization of three different dynamics regimes. At low frequencies (e.g. at 3 GHz, as the definition of “low” and “high” frequencies is here conditional), there is a quasi-periodic evolution between a dominant superconducting state with vortex chains at *U*(*t*) minima (not shown) and a significantly suppressed superconducting state (1) with a complex configuration of phase-slip regions (4) at *U*(*t*) maxima. The relatively high voltage (7) with a dominant peak at the ac frequency (10) is due to the normal regions.

At higher frequencies (e.g. at 6 GHz), complicated dynamics (8) exhibits a superposition of relatively fast oscillations mainly at the ac frequency and relatively long ($\sim 10\,\hbox {ns}$) intermittencies between the regime with a dominating superconducting state and a lower induced voltage around 1.2 mV and the regime with a suppressed superconducting state accompanied by phase-slip events (5) and a higher induced voltage around 3.2 mV. Under conditions of a very fast motion of vortices/antivortices, because of the retarded relaxation of quasiparticles outside the vortex cores, the emerging vortex/antivortex chains with a very weak modulation of the order parameter become indistinguishable from phase-slip lines^29,37^. Note that islands of quasi-1D-superconductivity are present near the slit banks (2). The FFT spectrum (11) reveals a rich set of low-frequency components. The highest peak occurs at zero frequency. The second- and third- highest peaks at $\sim 0.1 \,\hbox {GHz}$ and $\sim 0.25\,\hbox {GHz}$ are indicated by arrows in the inset of panel (11).

In the high-frequency regime (e.g., at 10 GHz), there is a quasi-periodic evolution of the dominating superconducting state both, at minima (not shown) and maxima (3) of *U*(*t*) with vortex chains close to the banks of the slit (6). The relatively low voltage (9) with a dominant peak at the ac frequency (12) is mainly induced in the narrow vicinity of the slit banks, where a normal-to-superconducting state conversion takes place.

### Effect of the dc magnitude at high ac frequencies

**Figure 6:**
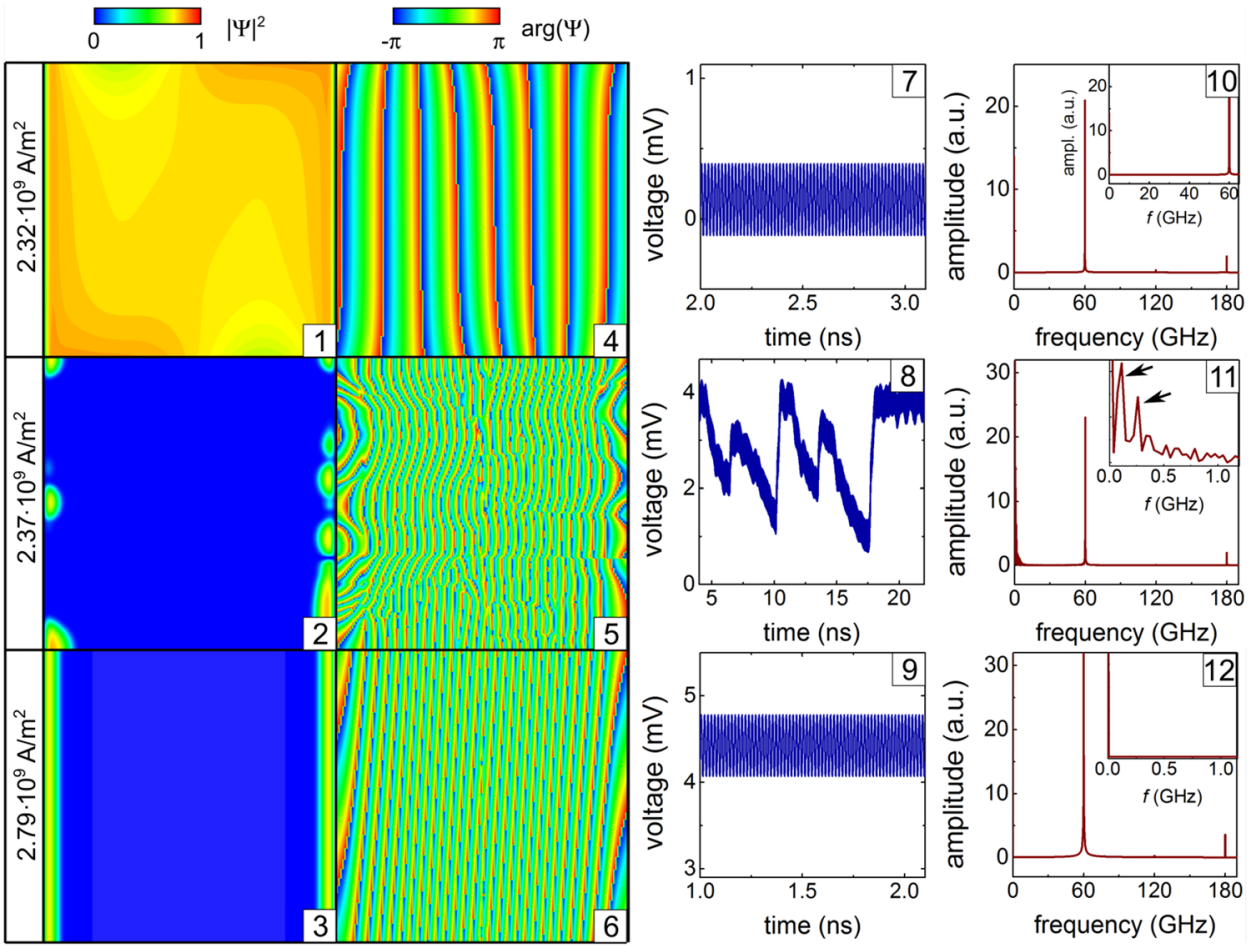
Effect of the dc magnitude on the superconducting transition. Modulus (panels 1–3) and phase (panels 4–6) of the order parameter at the maxima of time-dependent voltage (panels 7–9) and its FFT spectrum (panels 10–12) at $B = 2\,\hbox {mT}$, $f = 60\,\hbox {GHz}$ and $j_1/j_0 = 0.5$ for the dc densities $j_0$ indicated in panels (1)–(3). The second- and third-highest peaks are indicated by arrows in the inset of panel (11).

With increase of the dc current density at $f = 60\,\hbox {GHz}$ (Fig. 6), a transition occurs from the superconducting state filling almost the entire open tube typical of smaller dc densities (1) to the significantly suppressed superconducting state (2, 3), which is first accompanied by phase-slip events (5). (Note that $f=60\,\hbox {GHz}$ is still notably smaller than the gap breakdown frequency in Nb at $0.77T_{\mathrm {c}}$.) A transition between those regimes occurs between $j_0=2.32\,\hbox {GAm}^{-2}$ (7) and $j_0=2.34\,\hbox {GAm}^{-2}$ (8) when the dynamics of the induced voltage reveals an instability between the high values, typical of a stronger dc (9), and the low values, typical of weaker dc (7). Under this transition, the FFT spectrum experiences a dramatic change, exhibiting an abrupt increase of the dc voltage $U_0=0.33\,\hbox {mV}$ [decrease of the voltage modulation depth $U_1/U_0=0.7$] (7) to $U_0=2.24\,\hbox {mV}$ [to $U_1/U_0 =0.14$] (8). This transition opens up a novel way to experimentally unveil the otherwise unachievable patterns of the order parameter through observation of the time-dependent induced voltage in curved superconductor nanoarchitectures.

The FFT spectrum (11) reveals a rich set of low-frequency components. The highest peak occurs at zero frequency. Interestingly, the second- and third- highest peaks at $\sim 0.1\, \hbox {GHz}$ and $\sim 0.25\,\hbox {GHz}$, which are indicated by arrows in the inset of panel (11), are close to those in the FFT spectrum of the induced voltage for $f=6\,\hbox {GHz}$ in Fig. 5 (11). This fact implies that the low-frequency components of *U*(*t*) are due to the internal dynamics of the order parameter, which might be only weakly interplaying with the dynamics induced by the ac modulation. The time evolution of the order parameter and the induced voltage at the ac frequency $f = 60\,\hbox {GHz}$ is illustrated in Supplementary Video 3.

## Discussion

Our modeling reveals that the evolution of superconductivity in open nano/microtubes in an orthogonal-to-tube-axis magnetic field under a modulated (dc+ac) transport current manifests a plethora of inhomogeneous states. The key effect is a transition between two regimes in the superconducting dynamics. The first regime is characterized by a pronounced first harmonic in the FFT spectrum of the induced voltage at the frequency of the ac current. It is typical of two limiting cases, when the dominant area of the open tube is superconducting at relatively low magnetic fields and/or weak dc currents or normal at relatively high magnetic fields and/or strong dc currents. The second regime is represented by a rich FFT spectrum of the induced voltage with (i) pronounced low-frequency components due to the internal dynamics of superconducting vortices, phase-slip regions and superconducting screening currents and (ii) multiple harmonics of the ac frequency. This finding implies the possibility to experimentally unveil the distributions of the order parameter through observation of the time-dependent induced voltage and to control the modulated transport in superconductor nano/microarchitectures.

The applicability of the model of 2D superconductor micro/nanoarchitectures analyzed in the present paper is highly realistic, because such structures can be fabricated, e.g., from Nb^28^, Nb-C^32^ and W-C^31^. For instance, signatures of vortex and phase-slip patterns in nanohelices have been experimentally identified and supported by numerical simulations based on the TDGL equation^31^. In those structures, the occurrence of imperfections (mechanical defects in self-rolled films and impurity atoms in 3D-written structures) must be taken into consideration in the further research. Besides, real edge barriers for nucleation of superconducting vortices are not perfect (notches, materials composition variations etc). The quality of the barriers in superconductors is known to be decisive for the evolution of the order parameter in the entire sample^55^. However, the unveiled transitions between different configurations of topological defects governed by the global superconducting screening currents flowing over the entire structures are of topological nature and therefore are expected to be robust with respect to defects and impurities.

The dissipative nature of the transport of vortices and phase-slip regions, which induce a resistive state of micro/nanoarchitectures, raises an important task of heat removal, especially in the regime of close-to-depairing transport currents. Solution to this problem can be twofold: (i) by adding a shunt resistance with $R< R_{\mathrm {tube}}$ parallel to the open tube^44^ or (ii) embedding the open tube directly into liquid helium. Among challenges to be met in further work, there is a theoretical and an experimental one. The theoretical challenge is related to the (dc+ac)-driven escape of quasiparticles from the vortex cores^56^, leading to the complex dynamics of vortices in a quasiparticle “cloud” in the form of additional phase-slip lines^36,38^. The experimental challenge is associated with spurious capacitances/inductances in the transmission line, which may significantly modify the overall shape of the observed *U*(*t*), yet the FFT first-harmonic peaks should be expected to be clearly seen.

## Conclusions

In conclusion, we have investigated the dynamics of topological defects (vortices and slips of the phase) of the order parameter in (dc+ac)-driven open superconductor nanotubes. Relying upon the TDGL equation, we have revealed novel patterns of topological defects, which include phase-slip regions extending *along* the transport current direction, their *branching* and *coexistence* with Abrikosov vortices. We have identified two qualitatively different regimes in the voltage response which can be accessed experimentally. The first regime is characterized by a pronounced first harmonic in the FFT spectrum of the induced voltage. This regime occurs when the dominant area of the open tube is in the superconducting or normal state. The second regime features a rich FFT spectrum of the induced voltage, because of the complex interplay between the dynamics of vortices, phase-slip regions and superconducting screening currents. Our findings shed light on the spatiotemporal evolution of the superconducting order parameter in open nanotubes and allow for its control via the induced voltage. The topological transitions between vortex- and phase-slip-based transport regimes in curved micro/nanoarchitectures open up a possibility to efficiently tailor the superconductor’s voltage response via the 3D geometry and the topology of superconducting screening currents.

## Methods

### Numerical modeling

The superconducting state of the Nb tube with parameters stated in Table 1 is described by the TDGL equation for the complex-valued order parameter $\psi$^60,62^ in the dimensionless form

1 $$\begin{aligned} \frac{\partial \psi }{\partial t} = \left( \frac{\nabla }{\kappa } - i {\mathbf {A}} \right) ^2 \psi + (1 - |\psi |^2)\psi - i \kappa \varphi \psi , \end{aligned}$$

where $\varphi$ is the electric scalar potential. The boundary conditions

2 $$\begin{aligned} (\nabla - i {\mathbf {A}})\psi |_{n,{\mathrm {boundary}}} = 0 \end{aligned}$$

imply zero value of the normal component of the superconducting current at the edges of the system without electrodes. The scalar potential $\varphi$ is found as a solution of the Poisson equation coupled to the TDGL equation

3 $$\begin{aligned} \Delta \varphi = \frac{1}{\sigma }(\nabla , {\mathbf {j}}_{\mathrm {sc}}), \end{aligned}$$

where the superconducting current density is defined as ${\mathbf {j}}_{sc}= \frac{1}{2i\kappa }\left( \psi ^*\nabla \psi -\psi \nabla \psi ^*\right) - {\mathbf {A}}|\psi |^2$ and $\sigma$ is the normal conductivity. The transport current density $j_{tr}\left( y\right) =const\equiv j_{tr}$ is imposed via the boundary conditions for Eq. (3) at the edges, to which electrodes are attached $\left( {\mathbf {n}},\nabla \right) \varphi |_{\mathrm {electrode}} = -\left( \frac{1}{\sigma }\right) j_{\mathrm {tr}}$. The transport current density is modulated by the ac component with the frequency *f*

4 $$\begin{aligned} j_{\mathrm {tr}}(t) = j_0 + j_1\sin (2\pi ft). \end{aligned}$$

The vector potential components $A_s(s,y)$ and $A_y(s,y)$ (where $s=R\theta$) are chosen in the Coulomb gauge: $A_s(s,y)=0$; $A_y(s,y) = BR\cos \left( \frac{s}{R}\right)$. The set of Eqs. (1) and (3) is solved numerically, based on the link variables technique^62^. The relaxation method is used with a random initial distribution $\psi (s,y)$ of the order parameter. In the presence of transport current and magnetic field exceeding the lower critical field ($B>B_{\mathrm {c1}}$), the order parameter evolves to a quasi-stationary state, which is characterized by the quasi-periodic vortex nucleation/denucleation at the edge domains with the highest/lowest value of the normal to the surface component of magnetic field^62–65^ or the quasi-periodic occurrence of phase-slip events^29^. Vortices are moving paraxially along the tube and generate an electric field which is directed oppositely to the transport current density^23^. Finally, the average induced voltage *U*(*t*) is obtained by averaging the local difference of the values of the scalar potential $\varphi$, which are calculated at both slit banks at a given coordinate *y* in the paraxial direction, over the electrode length *L*

5 $$\begin{aligned} U(t) = \frac{1}{L}\int _0^L \left[ \varphi (\delta /2,y,t - \varphi (2\pi R- \delta /2,y,t)\right] dy, \end{aligned}$$

where $\delta$ is the slit width.

**Table 1:** Physical and geometrical parameters of the Nb films used for simulations.

Parameter	Denotation	Value	References
London penetration depth at $T=0$	$\lambda _0$	43 nm	^57^
Pippard coherence length	$\xi _0$	312 nm	^57^
Relative temperature	$T/T_{{\mathrm {c}}}$	0.77	With $T_{{\mathrm {c}}} \sim 9\,\hbox {K}$, $T \sim 7\,\hbox {K}$ is typical of experiment^58^
Resistivity	$\rho _0$	$70.2\,\upmu \Omega \hbox {cm}$	^57^
Electron mean free path	$l = 3.72\times 10^{-6}\,\upmu \Omega \hbox {cm}^2$/$\rho _0$	0.53 nm	Calculated after^59^ using $\rho _0$
Penetration depth	$\lambda =\lambda _0\sqrt{\frac{\xi _0}{2(1-T/T_c)\times 1.33l}}$	$1.3\,\upmu \hbox {m}$	Calculated after^60^ using $\lambda _0$, $\xi _0$, *l* and $T/T_{{\mathrm {c}}}$
Coherence length	$\xi =0.855\sqrt{\frac{\xi _0l}{1-T/T_c}}$	23 nm	Calculated after^60^ using $\xi _0$, *l* and $T/T_{{\mathrm {c}}}$
GL parameter	$\kappa = \lambda /\xi$	58	Calculated using $\lambda$ and $\xi$
Fermi velocity	$v_F$	$6\times 10^{5}\,\hbox {m}\,\hbox {s}^{-1}$	^61^
Film thickness	*d*	50 nm	^52,53^
Diffusion coefficient	$D = l v_F/3$	$1.06\times 10^{-4}\,\hbox {m}^2\,\hbox {s}^{-1}$	Calculated using *l* and $v_F$
Normal conductivity	$\sigma =l/\rho _0$	$1.42 (\upmu \Omega {{\mathrm {m}}})^{-1}$	Calculated using $\rho _0$

## Supplementary Information

Supplementary Video 1.

Supplementary Video 2.

Supplementary Video 3.

Supplementary Information.
