# Supplementary material for: Topological transitions in ac/dc﻿-driven superconductor nanotubes
Source: Sci Rep. 2022 Jun 16;12:10069. doi: 10.1038/s41598-022-13543-0 (PMC9203797; doi:10.1038/s41598-022-13543-0)
Supplement: Supplementary file 4 — Supplementary Information. [file 41598_2022_13543_MOESM4_ESM.pdf]

Supplementary information for the manuscript

Topological transitions in ac/dc-driven open superconductor nanotubes

Vladimir M. Fomin[1,2,3*], Roman O. Rezaev[4], Oleksandr V. Dobrovolskiy[5]

[1] *Institute for Integrative Nanosciences, Leibniz IFW Dresden, Helmholtzstraße 20, D-01069 Dresden, Germany*

[2] *Laboratory of Physics and Engineering of Nanomaterials, Department of Theoretical Physics, Moldova State University, strada A. Mateevici 60, MD-2009 Chisinau, Republic of Moldova*

[3] *Institute of Engineering Physics for Biomedicine, National Research Nuclear University "MEPhI", Kashirskoe shosse 31, 115409 Moscow, Russia*

[4] *Tomsk Polytechnic University, Lenin av. 30, 634050 Tomsk, Russia*

[5] *SuperSpin Lab, Nanomagnetism and Magnonics, Faculty of Physics, University of Vienna, Währinger Str. 17, 1090 Vienna, Austria*

* E-mail: v.fomin@ifw-dresden.de

Supplementary Video 1. Topological transitions in open superconductor nanotubes under a modulated transport current. 1. Evolution of the modulus and phase of the order parameter, the induced voltage and scalar potential for the different magnetic fields B and the dc+ac transport current modulation depth 0.5 at f = 0.6 GHz for open Nb nanotubes with length L=5 μm and radius 400 nm. The sizes of all panels (height × width) are equal to $L \times 2\pi R$ (length × circumference of the tube).

Supplementary Video 2. Topological transitions in open superconductor nanotubes under a modulated transport current. 2. Evolution of the modulus and phase of the order parameter, the induced voltage and scalar potential for the magnetic field B=4 mT and the dc+ac transport current with different values of the modulation depth at f = 0.6 GHz for open Nb nanotubes with length L=5 μm and radius 400 nm. The sizes of all panels (height × width) are equal to $L \times 2\pi R$ (length × circumference of the tube).

Supplementary Video 3. Topological transitions in open superconductor nanotubes under a modulated transport current. 3. Evolution of the modulus and phase of the order parameter, the induced voltage and scalar potential for the magnetic field B=2 mT and the dc+ac transport current with different values of the dc magnitude of the transport current density at the modulation depth 0.5 and f = 60 GHz for open Nb nanotubes with length L=5 μm and radius 400 nm. The sizes of all panels (height × width) are equal to $L \times 2\pi R$ (length × circumference of the tube).

Table SI-1. Parameters of the numerical simulations for open tubes with $R = 400$ nm, $L = 5$ μm

Sequence number	B, mT	j_0, GAm^{-2}	f, GHz	$\frac{j_1}{j_0}$	$\frac{T}{T_c}$	$U_0, \mu V$	$\frac{U_1}{U_0}$	$\frac{U_2}{U_0}$	$\frac{U_3}{U_0}$
**1**	0	2.1048	0.6	0.5	0.77	1346	1.127	0.118	0.094
**2**	2	2.1048	0.6	0.5	0.77	1318	0.985	0.172	0.068
**3**	2	2.1048	3.0	0.5	0.77	1892	0.412	0.072	0.051
**4**	2	2.1048	6	0.5	0.77	995	0.512	0.098	0.030
**5**	4	2.1048	0.6	0.3	0.77	998	1.111	0.146	0.103
**6**	4	2.1048	0.6	0.5	0.77	1474	1.046	0.035	0.131
**7**	4	2.1048	0.6	0.8	0.77	2056	1.053	0.033	0.093
**8**	4	2.1048	0.6	1.0	0.77	2163	1.143	0.073	0.111
**9**	6	2.1048	0.6	0.5	0.77	2026	0.730	0.066	0.086
**10**	10	2.1048	0.6	0.5	0.77	1772	0.796	0.081	0.066
**11**	2	2.4556	60	1.0	0.77	3750	0.295	0.001	0.036
**12**	6	2.4556	60	1.0	0.77	3826	0.289	0.001	0.035
**13**	10	2.4556	60	1.0	0.77	3750	0.314	0.000	0.038
**14**	0	2.1048	60	0.5	0.77	147.1	1.444	0.093	0.093
**15**	2	2.1048	60	0.5	0.77	149.7	1.008	0.074	0.074
**16**	4	2.1048	60	0.5	0.77	153.4	1.286	0.110	0.109
**17**	6	2.1048	60	0.5	0.77	171.3	1.213	0.093	0.093
**18**	10	2.1048	60	0.5	0.77	256.6	0.621	0.050	0.050
**19**	2	2.280	60	0.5	0.77	255,0	1.018	0.016	0.062
**20**	2	2.324	60	0.5	0.77	330.1	0.696	0.011	0.068
**21**	2	2.368	60	0.5	0.77	2243	0.142	0.001	0.011
**22**	2	2.412	60	0.5	0.77	2427	0.123	0.001	0.008
**23**	2	2.1048	1	0.5	0.77	1274	1.014	0.071	0.097
**24**	2	2.1048	10	0.5	0.77	237	1.073	0.194	0.082
**25**	2	2.1048	20	0.5	0.77	332	0.550	0.030	0.028
